# Isolation and Genomic Analysis of an Intracellular Mycobacterium gordonae from a Free-Living *Acanthamoeba* sp. in a Hospital Environment in Lima, Peru

**DOI:** 10.1128/mra.00784-22

**Published:** 2022-10-17

**Authors:** Alfonso M. Cabello-Vílchez, Alejandra Dávila-Barclay, Pablo Tsukayama

**Affiliations:** a Instituto de Medicina Tropical Alexander von Humboldt, Universidad Peruana Cayetano Heredia, Lima, Peru; b Laboratorio de Protistas Patógenos, Universidad Privada Norbert Wiener, Lima, Peru; c Laboratorio de Genómica Microbiana, Facultad de Ciencias y Filosofía, Universidad Peruana Cayetano Heredia, Lima, Peru; d Parasites and Microbes Programme, Wellcome Sanger Institute, Hinxton, United Kingdom; University of Rochester School of Medicine and Dentistry

## Abstract

Mycobacterium gordonae is a nontuberculous mycobacterium found in diverse environments and is considered an opportunistic pathogen in immunocompromised patients. We report the draft genome sequence of a Mycobacterium gordonae strain isolated from a free-living amoeba found in a nosocomial environment in Lima, Peru.

## ANNOUNCEMENT

Mycobacterium gordonae is an opportunistic nontuberculous mycobacterium (NTM) ubiquitous in water systems (1–4), where it shares a habitat with free-living amoebae (FLA) (5–8). Infections are associated with immunocompromised patients but may occur in immunocompetent hosts (9–15). Exposure to water networks and bodies poses a potential risk for disease (16, 17).

**FIG 1:**
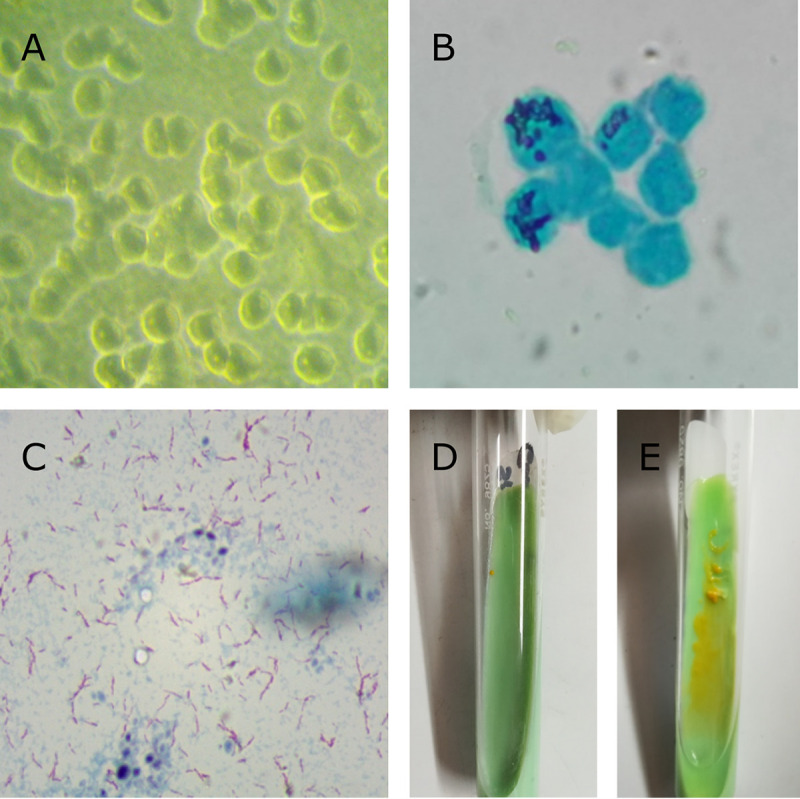
(A) Acanthamoebal trophozoites (phase-contrast microscopy, ×60 magnification). (B) Acanthamoebal cysts with acid-fast bacillus microcolonies, Ziehl-Neelsen stain (light microscopy, ×40 magnification). (C) Positive Ziehl-Neelsen-stained bacillus (light microscopy, ×100 magnification). (D) Single orange nontuberculous mycobacterium colony in Löwenstein-Jensen medium. (E) Restreaked mycobacterial colony in Löwenstein-Jensen medium.

Previous reports have shown that NTM can associate with amoeba hosts in the environment (5, 8, 18). Mycobacterium gordonae isolate NTM676 was found intracellularly in an amoebal cyst previously isolated from a faucet surface biofilm in a tertiary-level hospital in Lima, Peru. After 48 h of incubation at room temperature, a positive *Acanthamoeba* sp. culture grown in nonnutritive agar with a lawn of Escherichia coli ATCC 25922 was observed under phase-contrast microscopy (Fig. 1A). Page’s amoeba saline solution was added to the plate for 1 h and then streaked into a 15-mL conical tube. The centrifuged sediment was placed in a new tube and left at room temperature for 30 days. Aliquots were taken weekly for Ziehl-Neelsen staining.

We observed a slow-growing intracellular acid-fast bacillus (AFB) at week 4 inside amoebal trophozoites and cysts (Fig. 1B). A gentamicin and streptomycin solution (300 μg/mL) was added to the tube and incubated at room temperature until trophozoites were no longer present (Fig. 1C). The remaining cysts containing AFB were cultured in nonnutritive agar until trophozoite transformation and then lysed with sodium dodecyl sulfate (SDS) and decontaminated using modified Petroff’s method (19). The sediment was washed with phosphate buffer and inoculated in Löwenstein-Jensen (LJ) medium at 30°C for 25 days. A single small smooth orange scotochromogenic colony suggested the growth of an NTM-like organism (Fig. 1D). Further screenings were not conducted prior to whole-genome sequencing.

DNA was extracted from colonies restreaked in LJ medium (Fig. 1E) using the GeneJET DNA purification kit (Thermo Fisher Scientific, Waltham, MA). Genomic libraries were prepared using the Nextera XT kit (Illumina, San Diego, CA) and sequenced on an Illumina MiSeq instrument and a V2 kit, generating 2,082,926 250-bp paired-end raw sequences. Reads were quality checked and trimmed with FastQC v0.11.9 (20) and Trimmomatic v0.39 (21), removing reads shorter than 20 bp. *De novo* assembly using SPAdes v3.15.4 (22) resulted in 314 contigs, a total length of 7,266,850 bp with an *N*_50_ of 42,384 bp, mean coverage of 51×, and a GC content of 66.2%.

Resistome prediction with CARD 3.2.3 RGI 5.2.1 (23) identified genes associated with resistance to fosfomycin (*murA*, mutation C117D), rifamycin (*rbpA*; *rpoB,* mutations D516G, H526T, L511R), and macrolides (*mtrA*) with a >90% identity threshold and aminoglycosides [*aac(2')-Ic*] with an >80% identity threshold. PathogenFinder v1.1 (24) indicated a 75% probability of being a human pathogen, matching with 25 known virulence protein families, including ESAT-6 and others related to the ESX-5 secretion system, with a median identity threshold of 93.5%. All programs were run on default parameters.

This draft genome sequence reveals the antibiotic resistance and pathogenic potential of a nontuberculous mycobacterium found endosymbiotically in a free-living amoeba from hospital water systems.

### Data availability.

The whole-genome shotgun project for M. gordonae NTM676 is available at DDBJ/ENA/GenBank under the accession number JANFXG010000000. Raw sequence reads are available in the Sequence Read Archive (SRA) under the accession number SRR14802570.
